# Does treatment of the laryngeal mucosa reduce dystonic symptoms? A prospective clinical cohort study of mannose binding lectin and other immunological parameters with diagnostic use of phonatory function studies

**DOI:** 10.1007/s00405-011-1898-y

**Published:** 2012-01-31

**Authors:** Mette Pedersen, Martin Eeg

**Affiliations:** Ear, Nose, Throat Clinic, The Medical Centre, Østergade 18, 3., 1100 Copenhagen, Denmark

**Keywords:** Larynx, Mucosa, Voice, Oedema, Phonatory function studies, High-speed digital imaging, Electroglottography, Dystonia, Mannose binding lectin, Innate immune system

## Abstract

This study examined efficacy of the innate immune defence via the mannose binding lectin (MBL) in a cohort of 55 dystonic patients prospectively referred to the clinic with laryngeal mucosal complaints, who were placed on local steroids (budesonid inhaler, 400 μg 2 times daily) and antihistamines (fexofenadin 180 mg mostly 3 times daily) with adjuvant lifestyle corrections. Treatment efficacy of the larynx was assessed based on mucosal findings of the vocal folds examined with phonatory function studies (PhFS) comprising simultaneous high-speed digital images, kymography, electroglottography and voice acoustics combined with a visual score of arytenoids oedema, as these measures are indicative of the magnitude of laryngitis. Lactose and gluten intolerance and immunological analyses of the innate system were made systematically. Results showed that the genetic aspects of immunology did not reveal a role for the innate immune system, represented by the MBL. But an unexpected positive effect of the larynx treatment on dystonia symptoms was found evidenced by reduction of dystonic complaints and more normative results of PhFS, and a reduction of oedema of the inter arytenoids region. Symptoms relieve and better quality of life was observed on follow-up for the dystonia complaints.

## Introduction

Dystonia represents a neurological syndrome dominated by involuntary muscle contractions, with several kinds of dystonia recognised clinically. Theories of the development of dystonia include genetic- and immunological aspects and also acute provocations by, e.g. infection disorders. Specific stress provocations are seldom referred to in the literature. Syndromes of dystonia are clinically and genetically heterogenic disorders, the recent aspects include that the disorder can be reversible [1, 2].

Treatment options of dystonia are historically rarely documented in prospective randomised controlled studies, although a more constant symptom effect is obtained with the painful injections of neurotoxins with one product in an evidence-based study comparing two different products [3–7].

Treatments are often related to lifestyle [8]. Since 1976 treatment of laryngeal spasmodic dystonia is controlled by unilateral section of the recurrent nerve denervation either surgically or chemically. Through this approach understanding of laryngeal dystonia has been gained, and systematic diagnosis and treatment of spastic dysphonia was presented by Dedo and Izdebski [9, 10], yet, randomised controlled trials of treatment of spasmodic dystonia have not been made [11].

## Materials and methods

The 55 dystonia patients with an average age of 55 years were included prospectively in this observational cohort study [12] for 8 months The patients included 14 males (25.5%) and 41 females (74.5%) who had experienced dystonia-related symptoms of chronic laryngitis (mucosal symptoms) for an average of 13 years. The symptom duration time was between half a year and 42 years (Table 1). The localisation of the dystonia symptoms with laryngeal mucosal symptoms is presented in Table 2. Only 2 patients had genetic lactose intolerance and 1 had gluten intolerance out of the 55 dystonia patients. Out of nine patients examined for various dystonia genes 2 (22%) had DYT 1 genes. Botox had been given to all patients in the Danish hospital system. From the systematic blood test, 26 (47%) patients had a reduced function of the innate immune system (MBL <500 μg/L).

**Table 1:** Patient description

	Females (*N* = 41)	Males (*N* = 14)	Total (all patients) (*N* = 55)
Age (years)			
Mean	57.0	50.7	55.4
SD	12.9	16.1	13.9
Range	29–79	9–69	9–79
Symptom duration (years)			
Mean	11.7	15.9	12.7
SD	11.2	14.4	12.1
Range	0.5–50	3–41	0.5–50
MBL			
<500 μg/L	19 (46%)	7 (50%)	26 (47%)
>500 μg/L	17 (42%)	4 (29%)	21 (38%)
Missing	5 (12%)	3 (21%)	8 (15%)

**Table 2:** Number of patients in each subdivision of dystonia symptoms

Symptoms	1st consultation	2nd consultation after 2–3 weeks	
	*n* (%)	Average improvement (%)	Range (%)
Laryngeal	55 (100)		
Focal symptoms	36 (65)	40	0, 1–75
Segmental	18 (33)	20	0–50
Multifocal	21 (38)	31	0–50
General	26 (47)	60	0–98

Because the population described here presented symptoms of “laryngitis”, they were subjected to its treatment. Laryngitis complaints included: sore throat, dysphonia and mucosal complications. The intention was to evaluate an eventual immunological factor with the use of phonatory function studies (PhFS).

To assure objectivity of treatment efficacy, specific objective techniques (PhFS) capable of describing changes within vocal folds were employed. These included, high-speed digital imaging of the vocal folds, kymography, EGG and acoustics [13–18].

At the first examination, the duration and specific symptoms of laryngitis and the dystonia syndrome were discussed with the patients. Findings were stored in the data file and included visual scores of vocal folds regularity and abnormal inter arytenoids oedema on high speed digital images (HSDI), as earlier described [18], 1 being normal and 5 being maximal oedema closing the glottis. A plan for life style correction, local steroid inhaler of budesonid in the upper airways and the antihistamine fexofenadin was made. Medication of local steroids (budesonid inhaler, 400 μg 2 times daily) and antihistamines (fexofenadin 180 mg mostly 3 times daily) as well as lifestyle correction was given, based on mucosal findings. Lifestyle correction advice was related to upper airway infections, allergies and laryngo-pharyngeal reflux.

Physiologic adductor mobility of the vocal folds obtained from high-speed recordings were measured at three locations, corresponding to classical demarcation lines used in vocal folds segmentation: front, centre and rear part of the vocal cords during intonation, and the acoustical measures based on the high-speed films were performed. This is illustrated in Fig. 4. Measurements included quantitative measurement of the area between the vocal folds in the front, middle and rear parts. Qualitative evaluation of the regularity of vocal folds mobility and motility were assessed using kymography, electroglottography (EGG) and acoustics obtained simultaneously.

The multivariate voice analyses (Laryngograph Ltd.) were made combined with the high-speed images, frequency and intensity measures of a sustained tone/ah/as well as reading of a standard phonetically balanced text (“The Northern Wind and the Sun”). Electroglottography of the closed phases of the vocal cords vibratory cycle was calculated in a standard manner.

All measurements were repeated 2–3 weeks later. Follow-up was conducted 6–12 months later with a questionnaire sent to the patients to establish if the improvement was sustained.

## Results

In general, all 55 dystonic cases in the prospective cohort study of the dystonic patients showed elimination or significant clinical reduction of symptoms for “laryngitis”. Most surprisingly, we observed over the course of treating these 55 cases that all consecutive cases showed various responses with regard to their underlying aetiology, namely that dystonia severity was affected as a function of this treatment of the upper respiratory component, the larynx, including the vocal folds.

**Fig. 1:**
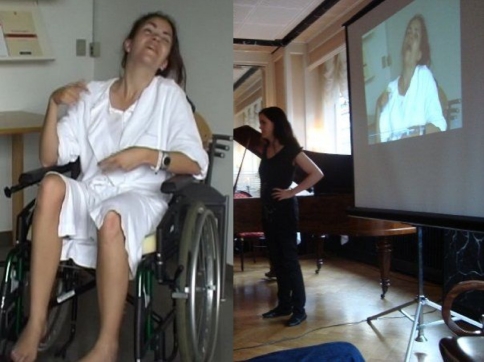
The first patient before and after treatment

Our first dystonia patient (Fig. 1) where high-speed images were used to document changes showed total elimination of universal dystonia symptoms. In addition, PhFS showed regularity of laryngeal actions. The elimination of dystonia in the larynx and in other parts of the body in some of these patients suggested a relationship between immunological deficiencies of the upper airways and the nature of dystonia, with relapses when the medication was terminated (Tables 1, 2).

What can account for this? Fexofenadin works as an anti inflammatory agent influencing the brain. Local steroids (budesonid) also work in an anti inflammatory way. A focus was also placed on the innate immune system, mannose binding lectin (MBL) since this is a very active agent in the early defence of the mucosa [19–23].

The overall high-speed images measures of the open quotients between the vocal cords are presented at the first and second examination in Table 3, second examination conducted 2–3 weeks later. The change in closure was significant in front and the middle part of the vocal cords from the measures for patients over time.

**Table 3:** High-speed films and MDVP results statistical analysis

	1st consultation			2nd consultation after 2–3 weeks			Change (2nd−1st consultation)			
	*N*	Mean	Std	*N*	Mean	Std	*N*	Mean	Std	*p* value
Open quotient front										
All dystonia patients	53	0.51	0.29	44	0.60	0.28	44	0.11	0.36	0.014*
MBL <500 μg/L	25	0.53	0.30	20	0.60	0.27	20	0.08	0.32	
MBL >500 μg/L	21	0.54	0.27	17	0.57	0.34	17	0.05	0.40	
MBL <500 μg/L versus MBL >500 μg/L										0.80^a^
Open quotient middle										
All dystonia patients	53	0.51	0.26	44	0.60	0.26	44	0.09	0.34	0.023*
MBL <500 μg/L	25	0.50	0.24	20	0.60	0.24	20	0.09	0.34	
MBL >500 μg/L	21	0.56	0.27	17	0.61	0.31	17	0.06	0.34	
MBL <500 μg/L versus MBL >500 μg/L										0.99^a^
Open quotient rear										
All dystonia patients	53	0.59	0.26	44	0.59	0.30	44	−0.02	0.37	0.65
MBL <500 μg/L	25	0.58	0.22	20	0.63	0.28	20	0.02	0.32	
MBL >500 μg/L	21	0.66	0.28	17	0.59	0.32	17	−0.10	0.40	
MBL <500 μg/L versus MBL >500 μg/L										0.58^a^
MDVP (reading)										
Reading variation										0.17
Frequency%	32	17.65	17.31	15	11.14	6.18	12	−2.45	10.54	
Reading intensity%	32	15.73	6.02	15	18.54	7.34	12	3.67	7.36	0.13
Reading Qx%	32	46.19	7.42	15	46.97	6.24	12	−0.55	5.79	0.73
MDVP (sustained tone)										
Tone Jitter%	31	6.97	14.49	15	4.07	5.30	11	0.70	5.46	0.68
Tone Shimmer%	31	13.20	13.57	15	9.70	9.24	11	−0.77	7.46	0.75
Tone Qx%	31	47.85	11.04	15	49.93	10.61	11	3.24	5.86	0.11

* Statistically significant on a 5% significance level

^a^Test in the linear statistical model where MBL is included as a fixed effect and baseline is included as a covariate

The oedema of the arytenoids regions was statistically significantly reduced (*p* = 0.0003), but with no difference between normal versus abnormal MBL values as seen in Table 4.

**Table 4:** High-speed films inter-arytenoid region oedema statistical analysis

	1st consultation			2nd consultation			Change (2nd–1st consultation)			
	*N*	Mean	Std	*N*	Mean	Std	*N*	Mean	Std	*p* value
All dystonia patients	55	2.71	0.60	49	2.35	0.63	49	−0.35	0.72	0.0003***
MBL <500 μg/L	26	2.69	0.62	22	2.32	0.57	22	−0.36	0.73	
MBL >500 μg/L	21	2.67	0.58	20	2.30	0.73	20	−0.40	0.75	
MBL <500 μg/L versus MBL >500 μg/L										0.90^a^

*** Statistically significant on a 0.1% significance level

^a^Test in the linear statistical model where MBL is included as a fixed effect and baseline is included as a covariate

**Fig. 2:**
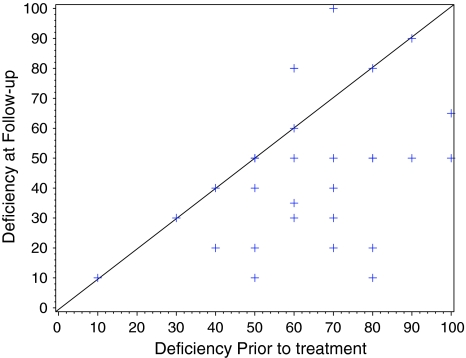
Mean change from prior assessment to follow-up assessment of −18.3 (*p* = 0.0001). 95% CI: [−27; −10]. 0 = no sickness, 100 = very sick

**Fig. 3:**
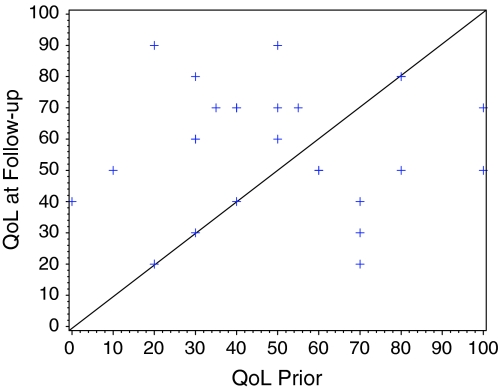
Mean change from prior assessment to follow-up assessment of 7.3 (*p* = 0.072). 95% CI: [−0.7; 15]. 0 = worst possible quality, 100 = best possible quality of life

The assessment of the severity of disease at the follow-up showed an improvement of 18.3 (*p* = 0.0001) and an improvement for the quality of life of 7.3 (*p* = 0.073) as seen in Figs. 2 and 3.

In Table 3, the acoustical measures before and after routine treatments of the patient’s complaint of symptoms of the larynx are presented. Notably, the electroglottographical measures showed no significance of change related to the upper airway mucosa, corresponding to the kymography measures in the HSDI. Other new measures of voice quality might show quantitative differences related to high-speed films of dystonia. Still no evidence exists till now.

The subdivisions of diagnoses of the dystonia cases were seen in Table 2. Mostly the shorter the duration of symptoms, the better effect of treatment of the upper airways was seen. The oedema was reduced of the arytenoids region in the back of the larynx with treatment of the upper airways. No difference was seen for the innate immune system component (MBL). The open quotients during intonation on high-speed films showed no difference between patients with abnormal MBL <500 μg/L and normal MBL >500 μg/L. The basic function of MBL in the innate immune system was illustrated in the book by Parham [22]. It attacks all pathogens coming into the body in a systematic way, very different from the adaptive defence system which does not seem to be involved here.

## Discussion

Randomised controlled studies are necessary in the future for various aspect of treatment of dystonia [12]. It was found in the presented prospective observational cohort study that the immune system in the dystonic population treated for symptoms of laryngitis complaints was optimised locally in the larynx, but also that some dystonia symptoms were reduced. This was a stunningly surprising outcome.

So, how can we account for this localised treatments effect on dystonic symptoms in this population? Although we tried to find detailed physiological and pharmacological documentation of the traditional medications used for dystonia, none had the sufficient evidence normally required in the Cochrane library, positively and negatively. In our study, the MBL part of the immune system did not seem to be involved. HSDI were useful to show better laryngeal function after treatment.

One possible explanation of effect is that the mucosa in the upper airways in the dystonia syndrome is related to neurological sensors in the mucosa, even if the dystonia is universal. Another speculation may be that the voice and the upper airway mucosa has a much more integrated function, not yet understood, for regulating the symptoms of dystonia. Repairing the histamine-related function in itself can have yet undefined results [24, 25].

One other possible explanation is that the dopamine D1 receptor is involved in voicing in animal experiments and ablation of D1 dopamine receptor—expressing cells, generates mice with seizures, dystonia, hyperactivity and impaired oral behaviour are shown in an animal study with advanced brain measures [26, 27]. A review of the neural control of vocalisation in mammals includes pharmacological activation of the neurotransmitter histamine stimulation in synapses in the periaqueductal grey [28].

With the clinically relevant improvement of the symptom status of dystonia of nearly 20% after 6–12 months, future studies on the immune system related to the effects of fexofenadin tablets and budesonid inhaler locally in the throat seem relevant to pursuit. With the traumas in the personal history of patients with the dystonia disorder even a small change of the quality of life must be noted.

**Fig. 4:**
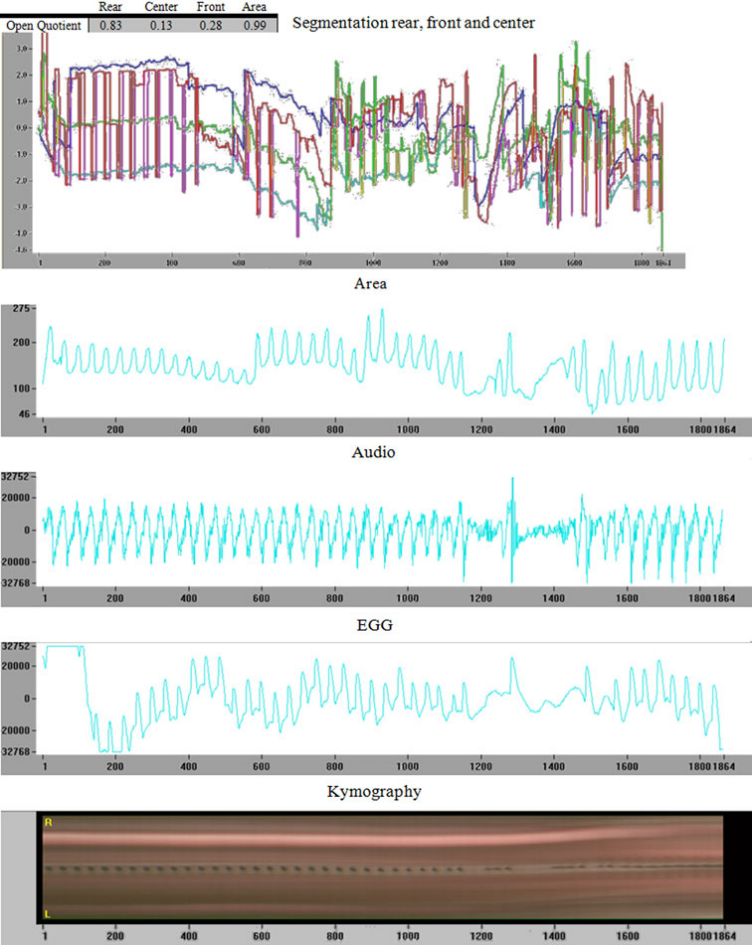
Segmentation curves. Segmentation curves for high-speed film calculations of open quotients in the front, center and rear parts of the vocal cords. Visual irregularities are illustrated due to a dystonia spasm—on movement curves of the vocal cords in front, center and rear, as well as area-, acoustical-, electroglottographical-, and kymographical curves

The high-speed images (HSDI) are interesting for the documentation of neurological deviations of the larynx because the true movement of the vocal cords and the laryngeal vestibule are captured (Fig. 4).

Scientific treatment documentation of the dystonia syndrome has mostly been carried out in animals. Careful evidence-based study designs are needed in the future as for study baselines, populations and statistical power calculations in humans. A supplementary study of antibodies has been suggested [29]. Central nucleus stimulation within the brain is a new treatment. Certainly, aspects of the immune system in the brain should be taken into account related to voicing, on an earlier stage, before operations on dystonia are considered [30]. Local immunological and eventual genetic aspects of the upper airways must be focused upon.

In summary, the results of this study indicates that the group of dystonia patients with low MBL (MBL <500 μg/L) responds to the treatment with local steroid inhaler of budesonid and antihistamine fexofenadin as well as the patients with normal levels of MBL. Other immune system-related factors should systematically be analysed in the future.
